# Safety standards and socioeconomic disparities in school playground injuries: a retrospective cohort study

**DOI:** 10.1186/1471-2458-10-542

**Published:** 2010-09-08

**Authors:** Alison K Macpherson, Jennifer Jones, Linda Rothman, Colin Macarthur, Andrew W Howard

**Affiliations:** 1 York University, 4700 Keele Street, Toronto ON, Canada; 2 McMaster University, 1280 Main Street, West Hamilton ON, Canada; 3 The Hospital for Sick Children, 555 University Avenue, Toronto ON, Canada; 4 Bloorview Kids Rehab, 150 Kilgour Road, Toronto ON, Canada

## Abstract

**Background:**

Playground injuries are fairly common and can require hospitalization and or surgery. Previous research has suggested that compliance with guidelines or standards can reduce the incidence of such injuries, and that poorer children are at increased risk of playground injuries.

**Objective:**

The objective of this study was to determine the association between playground injury and school socioeconomic status before and after the upgrading of playground equipment to meet CSA guidelines.

**Methods:**

Injury data were collected from January 1998-December 1999 and January 2004 - June 2007 for 374 elementary schools in Toronto, Canada. The objective of this study was to investigate the effect of a program of playground assessment, upgrading, and replacement on school injury rates and socio-economic status. Injury rates were calculated for all injuries, injuries that did not occur on equipment, and injuries on play equipment. Poisson regression was performed to determine the relationship between injury rates and school socio-economic status.

**Results:**

Prior to upgrading the equipment there was a significant relationship between socio-economic status and equipment-related injuries with children at poorer schools being at increased risk (Relative risk: 1.52 [95% CI = 1.24-1.86]). After unsafe equipment was upgraded, the relationship between injury and SES decreased and was no longer significant (RR 1.13 [95% CI = 0.95-1.32]).

**Conclusions:**

Improvements in playground equipment can result in an environment in which students from schools in poorer neighbourhoods are no longer at increased risk of injuries on play equipment.

## Background

Unintentional injuries are the leading cause of death and one of the leading causes of hospitalization for children over one year in Canada [1]. Sports and recreation injuries, including injuries on playgrounds, are a common cause of unintentional injury [2]. For example, over a one year period (2004-2005), 8,231 children 14 years of age and under in Ontario visited an emergency department because of a playground injury and of these, almost 6% required at least one night in hospital. More than half (58%) of those injured were between 5 and 9 years of age [3]. Previous studies have identified improving school playgrounds as a promising strategy to prevent these injuries [4,5]. Upgrading playground equipment may be one way to reduce the burden of playground injuries because injuries due to falls from equipment are more severe than other falls on the playground. One previous study reported that fractures from playground equipment falls are 3.9 times more likely to require hospitalization and or surgery than are fractures from standing height falls on the playground [6].

In general, childhood injuries vary by socioeconomic status (SES). Injury morbidity and mortality are strongly associated with factors such as poverty, single parenthood, low maternal education, poor housing, large family size and parental drug and alcohol abuse [7]. As SES declines, the risk of injury mortality increases [8-11] as does the rate of non-fatal injuries [12-17]. In some cases, this relationship may be linked to the environment in which children live. For example, children living in deprived neighborhoods have higher rates of injury than children from more affluent areas [15]. A previous Canadian study found that children with the lowest SES had 1.37 times higher rates of recreation and play injuries, compared to children with the highest SES [13]. The prevalence of playground hazards has been found to be higher in poorer areas [18,19].

In the summer of 2000, a program was initiated by the Toronto District School Board to upgrade school playground equipment throughout the city to comply with new Canadian Standards Association (CSA) standards for playground equipment that address equipment height, surfacing of playgrounds, entrapment angles, and other safety issues. Equipment that was deemed to be the most unsafe (potential for falls from heights greater than 2 metres, potential for entrapment, or non-absorbent surfacing) was removed immediately. Equipment that was thought to be able to be repaired was retrofitted. Schools then had the opportunity to replace the equipment, or to leave the schoolyards without new equipment. Subsequent investigation showed that compliance with the playground standards was associated with a reduction in playground injuries [20]. The objective of this study was to determine the association between playground injury and school SES before and after the upgrading of playground equipment to meet CSA guidelines.

## Methods

### Study population

Data from 374 elementary schools in Toronto, Canada were included in the study. These schools had a population of approximately 145,000 students per year over the study period, which included January 1998 to December 1999 for the period prior to replacement, and January 2004 to June 2007 for the period subsequent to replacement. Children who attended private schools and religious schools were not included in either the numerator or the denominator. The schools ranged in size, with the smallest including 62 students, the largest 1600. The average school population was 388 students.

### Study context

In 2000, the Toronto District School Board initiated a program of removal and replacement of playground equipment that did not comply with standards for playground equipment provided by the Canadian Standards Association. Much of the equipment was replaced by compliant equipment. The methodology is further described in previous research that suggested that the program was successful in reducing the risk of injury on school playgrounds [20].

### Defining SES: The Learning Opportunity Index (LOI)

The Learning Opportunity Index is used to define school SES by the Toronto District School Board. The LOI is calculated using the social and demographic characteristics of the student population based on data collected by Statistics Canada from the Canadian census. Data included in the LOI include family income, proportion of single parent families, housing (detached, apartment buildings), parental education, neighborhood immigration, the number of students at the school who arrived in Canada in the past 5 years, and records of student mobility [21]. The score is a weighted average of the variables over the three previous years: 50% for the current year, and 25% each for the previous two years [21]. The LOI score is used to classify each school in the school board. LOI scores range from 0 (wealthiest) to 0.97 (poorest). The average score is 0.53 with a standard deviation of 0.27. Schools with the highest LOI score receive support from the Ministry of Education's Learning Opportunities Grant to help equalize learning opportunities [21].

We used the LOI scores for 2001 and 2005 because they included the best available data for the years of our study (1998 to 2007). According to the school board, there is very little variability in LOI scores within schools from year to year, as suggested by the reported correlation of 0.986 between 2003 and 2005 LOI scores [21].

### Defining injuries within elementary schools

A database of incident reports from the Ontario School Board Insurance Exchange (OSBIE) was used to identify all injury events occurring in elementary schools (grades Junior Kindergarten to Grade 6 or 8) in the Toronto District School Board. Data were available for 20 months prior to the application of CSA standards, and 36 months subsequent to the application of CSA standards. The summer months of July and August were excluded because children in Canada do not attend school during these months. Information in the database is provided by school staff whenever an incident occurs on school property during school hours. The threshold for completing a report is "whenever medical or dental attention is required" and this includes injuries attended to by teachers or school staff as well as those in which the child went home or to a health facility. All students who were injured in the outdoor school area were included [e.g. the child was injured on the playground, field, or play equipment]. Injuries from the OSBIE data were analyzed in three ways: all outdoor injuries, outdoor non-equipment injuries, and injuries occurring on play equipment. Any injury in the OSBIE data that were coded or described as occurring on a slide, swing, climber, monkeybar, playscape, structure, or play equipment, were identified as "play equipment injuries", while injuries including playing soccer, running, or playing ball were classified as 'non-equipment injuries'.

### Statistical analysis

Injury rates were calculated using student months (student population times the number of school months) prior to and subsequent to the application of CSA standards. Student populations from 2001 (the earliest year for which population information was available) were used as the denominator for injury rates for the period prior to application. Student populations from 2006 were used as the denominator for the period subsequent to the upgrading of playground equipment. Poisson regression was used to determine the relationship between the rate of playground injuries and LOI scores for both time periods. Statistical analyses were conducted using SAS version 9.1.

Ethical approval for the study was received from the research ethics review board at the Hospital for Sick Children, Toronto, Canada.

## Results

### Prior to the application of standards (January 1998-December 1999)

Prior to the application of the CSA standards a total of 5,378 injuries were reported by 364 schools. As shown in Table 1, the mean rate of all outdoor injuries was 2.04/1000 student months and ranged from 0.1 to 26.33. The mean rate of non-equipment injuries was 1.51/1000 (range from 0-26.43) and for equipment injuries was 0.50 (range from 0 to 4.82). The association between the school's LOI score and all playground injuries; both non-equipment and equipment are presented in Table 2. The relative risk of all outdoor injury with each unit increase in LOI score was 1.65 (1.50 -1.82). The corresponding rate for equipment-related injuries was 1.52 (1.24-1.86), indicating a socio-economic gradient for playground injuries.

**Table 1:** Injury rates in Toronto District School Board schools prior to and subsequent to the replacement of unsafe play equipment

		Prior to replacement	Subsequent to replacement
**All Outdoor Injuries**	Number of injuries	5378	8380
	Injury rate per 1000 student months (range)	2.04 (0.1-26.3)	1.76 (0.1-29.9)
Non-equipment injuries*	Number of injuries	4013	6791
	Injury rate per 1000 student months (range)	1.51 (0-21.7)	1.40 (0-26.4)
Equipment-related injuries*	Number of injuries	1272	1589
	Injury rate per 1000 student months (range)	0.50 (0-4.8)	0.36 (0-5.36)

* 93 children were missing values for non-equipment or equipment injury

**Table 2:** Relative risk of playground injury as school SES decreases, prior to and subsequent to equipment replacement (Poisson regression)

	Prior to replacement (RR*, 95% CI)	Subsequent to replacement (RR, 95% CI)
All Outdoor Injuries	1.65 (1.50-1.82)	2.07 (1.91-2.24)
Non-equipment injuries	1.68 (1.50-1.89)	2.41 (2.20-2.64)
Equipment-related injuries	1.52 (1.24-1.86)	1.13 (0.95-1.32)

* interpretation of the relative risk: With each unit decrease in school SES as measured by the Learning Opportunities Index, the relative risk of injury increases. For equipment related injuries prior to replacement, the relative risk increases 1.52 times with each unit decrease in school SES.

### Subsequent to Application of Standards (January 2004-June 2007)

A total of 8,380 injuries were reported by 374 elementary schools between January 2004 and June 2007. As shown in Table 1, the mean rate of total injuries occurring anywhere outside in the playground was 1.76/1000 student months (range 0.05 to 29.94). The mean rate of non-equipment injuries was 1.40/1000 student months (range from 0.00 - 5.26), and for playground equipment injures was 0.36 (range 0.00 - 26.43)

A significant relationship remained between a school's LOI score and the rate of total injuries, with a greater risk of injury in poorer schools. However, the association between SES and injuries that occurred on the play equipment subsequent to the upgrading of equipment was no longer statistically significant with a relative risk of 1.13 (95% CI 0.95-1.32).

## Discussion

This study examined the relationship between socioeconomic status and outdoor injury rates in elementary school children. A significant relationship was found between low SES and playground injury rates. However, subsequent to the modification of the play equipment, the relationship between LOI score and injury on play equipment was not statistically significant. Further, after the playgrounds were modified, the injury rate for play equipment dropped across all schools.

The initial association between SES and all outdoor injuries is supported by previous research related to unintentional childhood injuries and SES [10-16]. However, that there was no significant relationship between the rate of playground injuries that occurred on play equipment and SES status in schools subsequent to the modification of playgrounds may provide important insight into mechanisms for preventing childhood injuries. The play equipment is a controlled environment, and had been modified to meet CSA standards across all the schools in the school board. Our results suggest that the program to improve the play equipment appears to have been successful in equalizing the risk of injury on equipment between schools with different SES scores. This result supports the concept that modification of the built environment is a successful strategy to reduce inequity in childhood injury [22].

In general, the relationship between SES and injury is not consistent across different types of injury. Sports and recreation injuries are more common in children from a higher SES, perhaps because they have increased access to facilities and resources to be able to participate in these types of activities [12,13,23-26]. For example, one study found that children of higher SES had a higher injury rate from drowning, because their families were more likely to have a swimming pool at their house than children of a lower SES [23]. Our results cannot be explained by differential access to recreational equipment and facilities since all of the schools had play equipment available that was required to meet the same safety standards. However, it is possible that play equipment at poorer schools was of poorer quality prior to the upgrading of equipment.

Another methodological concern related to a lack a socioeconomic gradient in non-fatal injuries has been explained by claiming that low SES individuals may not have equal access to care and therefore, some non-fatal injuries go unreported [26]. If individuals do not have access to health care, or cannot afford it, they may be less likely to be included in the data for minor and less severe injuries [27-29]. However, this is unlikely to explain our findings because reporting was done by school staff with no consideration of whether the injury was treated at a health facility. Further, schools have standardized guidelines related to the reporting of incidents.

Although the playground injury rate for all injuries decreased subsequent to the upgrading of equipment, a socioeconomic gradient for non-equipment injuries remained. Of particular concern is the increased socio-economic disparity in non-equipment injuries. We hypothesize that the socioeconomic disparity in non equipment related injuries is explained by a difference across schools in playground environment outside the equipment area. Although the schools could not provide us with evidence related to differences between schools with different LOI scores, we believe that differences may be related to the availability of funds to upgrade the outdoor environment and the presence of adults on the playground. For example, schools in wealthier areas may have had the ability to raise funds to make improvements to fields, retaining walls, and other outdoor areas, whereas, poorer schools may have only upgraded the playground equipment. There may also be differences across schools in the presence of adults in the school yard. Parents in wealthier neighbourhoods may be more available to volunteer to oversee students during the midday break. These differences warrant further investigation.

There were several limitations to this study. First, the threshold for reporting injuries is fairly low and there may have been differential reporting of injuries between the schools. It is possible that there was a systematic tendency to report more superficial injuries in lower socioeconomic schools. However, all schools have the same guidelines for reporting an injury, so the variation by SES is not likely to be due to reporting differences. A second limitation is that the validity of the LOI score has not been extensively tested, although it is based on actual census data of the school catchment areas. Finally, it is difficult to determine whether changes in injury rates are a result of the intervention to upgrade playground equipment to a standard safety level throughout Toronto schools or whether other factors, such as increased teacher supervision, or differential exposure to playground equipment in schools with different SES contributed to the difference in injury rates. It is possible that children played less (or more) on equipment after it was replaced. Further studies in this area should include measures of exposure to play where possible.

A goal of the school board that participated in this study is to "identify and eliminate socio-economic class biases and barriers in Board policies guidelines, day-to-day operations, protocols, and practices" [30]. The results of this study suggest that one way to achieve this may be to equalize the risk of injury on playground equipment. The next injury prevention challenge for schools is to find methods to equalize the risk of injury in all areas of the playground. It will be important to identify appropriate strategies for reduction of non-equipment injuries. These strategies may include increased supervision, behavioral intervention programs, more structured activities, and changes to other aspects of the built environment. The school playground is an important learning environment for all children, and it is essential that children have equal opportunity for safe play in all school playgrounds.

## Conclusion

Upgrading of school playground equipment to meet CSA standards reduces the socio-economic gradient for this common cause of childhood injury.

## Competing interests

The authors declare that they have no competing interests.

## Authors' contributions

AKM, AWH, CM, and LR designed the study. JJ, LR, and AKM analyzed the data. AKM and JJ drafted the initial article. All authors were involved in critical evaluation and editing of the manuscript, and read and approved the final version.

## Pre-publication history

The pre-publication history for this paper can be accessed here:

http://www.biomedcentral.com/1471-2458/10/542/prepub

## References

[B1] Health Canada Injury surveillance in Canada: Current realities, challenges 2003

[B2] Canadian Institute for Health Information Over 8,500 playground injury-related ERvisits in Ontario, reports CIHI 2004

[B3] Canadian Institute for Health Information 23 children visit an Ontario ER daily due to playground injuries 2007

[B4] Phelan KJ Khoury J Kalkwarf HJ Lanphear BP Trends and patterns of playground injuries in United States children and adolescents [see comment] Ambulatory Pediatrics 2001 1 4 227 33 10.1367/1539-4409(2001)001<0227:TAPOPI>2.0.CO;2 11888406

[B5] Macarthur C Hu X Wesson DE Parkin PC Risk factors for severe injuries associated with falls from playground equipment Accid Anal Prev 2000 32 3 377 82 10.1016/S0001-4575(99)00079-2 10776854

[B6] Fiissell D Pattison G Howard A Severity of playground fractures: play equipment versus standing height falls Inj Prev 2005 11 337 339 10.1136/ip.2005.009167 16326766 PMC1730287

[B7] United Nations Children's Fund Innocenti Research Centre A League Table of Child Deaths by Injury in Rich Nations 2001 2 1 17

[B8] Roberts I Power C Does the decline in child injury mortality vary by social class? A comparison of class specific mortality in 1981 and 1991 BMJ 1996 313 784 786 8842070 10.1136/bmj.313.7060.784 PMC2352192

[B9] Birken CS Parkin PC To T Macarthur C Trends in rates of death from unintentional injury among Canadian children in urban areas: influence of socioeconomic status CMAJ 2006 175 8 867 868 16998078 10.1503/cmaj.051207 PMC1586087

[B10] Carey V Vimpani G Taylor R Childhood injury mortality in New South Wales: Geographical and socio-economic variations J Paediatr Child Health 1993 29 136 140 10.1111/j.1440-1754.1993.tb00466.x 8489794

[B11] Cubbin C LeClere FB Smith GS Socioeconomic status and injury mortality: individual and neighbourhood determinants J Epidemiol Community Health 2000 54 517 524 10.1136/jech.54.7.517 10846194 PMC1731715

[B12] Jolly DL Moller JN Volkmer RE The socio-economic context of child injury in Australia J Paediatr Child Health 1993 29 438 444 10.1111/j.1440-1754.1993.tb03016.x 8286160

[B13] Faelker T Pickett W Brison RJ Socioeconomic differences in childhood injury: a population based epidemiologic study in Ontario, Canada Inj Prev 2000 6 203 208 10.1136/ip.6.3.203 PMC1730634 11003186

[B14] Suecoff SA Avner JR Chou KJ Crain EF A comparison of New York City playground hazards in high- and low-income areas Arch Pediatr & Adolescent Med 1999 153 4 363 6 10.1001/archpedi.153.4.363 10201718

[B15] Cradock AL Kawachi I Colditz GA Hannon C Melly SJ Wiecha JL Gortmaker SL Playground safety and access in Boston neighborhoods Am J Prev Med 2005 28 4 357 63 10.1016/j.amepre.2005.01.012 15831341

[B16] Roberts I Marshall R Norton R Borman B An area analysis of child injury morbidity in Auckland J Paediatr Child Health 1992 28 438 441 10.1111/j.1440-1754.1992.tb02713.x 1466939

[B17] Durkin MS Davidson LL Kuhn L O'Connor P Barlow B Low-income neighborhoods and the risk of severe pediatric injury: a small-area analysis in northern Manhattan Am J Public Health 1994 84 4 587 592 10.2105/AJPH.84.4.587 8154561 PMC1614793

[B18] Haynes R Reading R Gale S Household and neighbourhood risks for injury to 5-14 year old children Soc Sci Med 2003 57 625 636 10.1016/S0277-9536(02)00446-X 12821011

[B19] Laing GJ Logan S Patterns of unintentional injury in childhood and their relation to socio-economic factors Publ Health 1999 113 291 294 10.1016/S0033-3506(99)00182-1 10637521

[B20] Howard AW Macarthur C Willan A Rothman L Moses-McKeag A Macpherson AK The effect of safer playground equipment on playground injury rates among children CMAJ 2005 172 11 1443 15911858 10.1503/cmaj.1041096 PMC557979

[B21] Toronto District School Board. Part 1 Elementary Learning Opportunities Index 2005 http://www.tdsb.on.ca/wwwdocuments/about_us/external_research_application/docs/loi%202004-5.pdf Accessed March/1, 2008

[B22] Howard AW Child Safety and the Built Environment CMAJ 2009 in press 19805499

[B23] Nixon J Pearn J An investigation of socio-demographic factors surrounding childhood drowning accidents Soc Sci Med 1978 12 387 390 705383

[B24] Ni H Barnes P Hardy AM Recreational injury and its relation to socioeconomic status among school aged children in the US Inj Prev 2002 8 60 65 10.1136/ip.8.1.60 11928978 PMC1730809

[B25] Simpson K Janssen I Craig WM Pickett W Multilevel analysis of associations between socioeconomic status and injury among Canadian adolescents J Epidemiol Community Health 2005 59 1072 1077 10.1136/jech.2005.036723 16286497 PMC1732968

[B26] Langley J Silva P Williams S Socio-economic status and childhood injuries Aust Paediatr J 1983 19 237 240 6673723 10.1111/j.1440-1754.1983.tb02110.x

[B27] Cubbin C LeClere FB Smith GS Socioeconomic status and the occurrence of fatal and nonfatal injury in the United States Am J Public Health 2000 90 1 70 77 10.2105/AJPH.90.1.70 10630140 PMC1446109

[B28] Williams JM Currie CE Wright P Elton RA Beattie TF Socioeconomic status and adolescent injuries Soc Sci Med 1996 44 12 1881 1891 10.1016/S0277-9536(96)00297-3 9194249

[B29] Reading R Langford IH Haynes R Lovett A Accidents to preschool children: comparing family and neighbourhood risk factors Soc Sci Med 1999 48 321 330 10.1016/S0277-9536(98)00311-6 10077280

[B30] Toronto District School Board Equity foundation statement and commitments to equity policy implementation 2000 http://www.tdsb.on.ca/wwwdocuments/programs/Equity_in_Education/docs/Equity_Foundation_Statement.pdf Accessed March/2, 2008

